# Commentary: Task-Switching in Pigeons: Associative Learning or Executive Control?

**DOI:** 10.3389/fpsyg.2017.01420

**Published:** 2017-08-22

**Authors:** Xiangqian Li, Bingxin Li, Martin Lages, Gijsbert Stoet

**Affiliations:** ^1^School of Psychology, University of Glasgow Glasgow, United Kingdom; ^2^School of Social Sciences, Leeds Beckett University Leeds, United Kingdom

**Keywords:** task switching, executive control, associative learning, monkey, pigeon

Switching between two tasks impairs human performance—resulting in what is known as task-switching costs (TSCs): Human participants perform more slowly and are more error-prone in switch trials compared to repeat trials (Vandierendonck et al., 2010). One of the most prominent theories, the task-set reconfiguration account suggests that switching to a new task-set involves a rule-based cognitive process (Monsell et al., 2003). Task-set reconfiguration requires executive control (EC) that is not necessary when repeating tasks. Although “task-set” and “EC” are not well-defined concepts, subjects must understand the task rules before they can reconfigure a task-set. Consequently, this account predicts that TSCs should disappear if subjects switch between tasks without applying task rules. In contrast to this prediction, it was found that bivalent stimuli triggered TSCs even when participants had no explicit understanding of the task rules (Forrest et al., 2014). This suggests that processes other than reconfiguration and EC contribute to TSCs.

Meier et al. (2016) put forward an alternative account. They addressed one of the disadvantages in previous studies. When task rules are not explicitly stated it was assumed that participants learned all the cue-stimulus combinations and their corresponding responses. It is also possible, however, that participants did not infer the task rules and that their performances were based on “associative learning”—gradually establishing stimulus-response mappings by trial and error. This would imply that no task-set reconfiguration and EC was involved in TSC. Meier et al. (2016) suggested that EC plays a role in task-switching even when researchers take precautions to eliminate task-set reconfiguration processes.

To address this issue, Meier et al. (2016) conducted a task-switching study with pigeons. They argued that pigeons do not have high-level cognition and EC, and therefore can only use associative learning to perform in a task-switching paradigm (but see Soto and Wasserman, 2010; Castro and Wasserman, 2016). In their study, the pigeons showed no increased error rates in switch trials. They concluded that TSC only emerge when human participants employ EC as in task-set reconfiguration.

There is a problem with the deductive reasoning by Meier et al. (2016). Studies on monkeys demonstrated that monkeys can switch between tasks without exhibiting TSC (Stoet and Snyder, 2003; Avdagic et al., 2014). However, there is clear evidence that monkeys employed some form of EC. Firstly, Avdagic et al. (2014) used the so-called “SimChain” paradigm. In this paradigm simple associative learning cannot explain the monkeys' behavior because the stimuli vary considerably across trials (for details see Jensen et al., 2013). Secondly, Stoet and Snyder (2003) reported that with ITIs as short as 170 ms, monkeys had small but significant TSCs. It is unlikely that the monkeys applied EC when the ITI was short but performed associative task switching when ITIs were longer. Instead we propose that the small TSCs may be due to interference between trials (Kiesel et al., 2010; Vandierendonck et al., 2010). Thirdly, neurophysiological results indicated that neurons in posterior parietal cortex of the monkey encoded task-set information independently of stimulus features (Stoet and Snyder, 2004).

Applying the same reasoning as Meier et al. (2016) to the results of studies in monkeys, we arrive at a different conclusion: Since monkeys can use EC without showing TSC, TSC in humans must be caused by factors other than EC. Given the mixed and incoherent evidence on this issue, it seems problematic to deduce the source of human TSC from studies in monkeys and pigeons. We speculate that there are at least two possible explanations that may have eliminated TSCs in pigeons and monkeys. Firstly, pigeons may have switched between tasks without employing EC. They simply recognized each stimulus and gave the associated response. Therefore, the difference in TSC between task-switching studies in humans and pigeons may reflect different cognitive default processes.

Another possibility is that human participants can eliminate distractions by focussing on single tasks when switching between different tasks (Stoet and Snyder, 2007, 2009). Although monkeys, and possibly pigeons, have some form of EC, they may not focus on a single task in the same way as humans. They may not employ the same cognitive functions and therefore exhibit different task-switching characteristics. For example, one study suggests that some of the human TSC are caused by passive interference from previous trials (Kiesel et al., 2010). It is possible that this interference is different for humans, monkeys, and pigeons. For example, interference in humans may fade away gradually whereas interference in monkeys and pigeons may decay more quickly.

Meier et al. (2016) made a valuable contribution by highlighting that humans have different TSC characteristics if they learn task switching by association. They also suggested that pigeons perceive the task cue and the target stimulus as a single compound stimulus whereas human participants cannot help but process cue and stimulus separately. We suggest that the discussion about the sources of TSC is far from settled, leaving open a number of avenues for investigation. EC and associative learning accounts seem too simplistic to explain different characteristics of TSC across species.

## Author contributions

All authors listed have made a substantial, direct and intellectual contribution to the work, and approved it for publication.

### Conflict of interest statement

The authors declare that the research was conducted in the absence of any commercial or financial relationships that could be construed as a potential conflict of interest.

## References

[B1] Avdagic E. Jensen G. Altschul D. Terrace H. S. (2014). Rapid cognitive flexibility of rhesus macaques performing psychophysical task-switching. Anim. Cogn. 17, 619–631. 10.1007/s10071-013-0693-0 24132412 PMC3988220

[B2] Castro L. Wasserman E. (2016). Executive control and task switching in pigeons. Cognition 146, 121–135. 10.1016/j.cognition.2015.07.014 26407340

[B3] Forrest C. L. Monsell S. McLaren I. P. (2014). Is performance in task-cuing experiments mediated by task set selection or associative compound retrieval? J. Exp. Psychol. Learn. Mem. Cogn. 40, 1002–1024. 10.1037/a0035981 24564543

[B4] Jensen G. Altschul D. Danly E. Terrace H. (2013). Transfer of a serial representation between two distinct tasks by Rhesus Macaques. PLoS ONE 8:e70285. 10.1371/journal.pone.0070285 23936179 PMC3729468

[B5] Kiesel A. Steinhauser M. Wendt M. Falkenstein M. Jost K. Philipp A. M. (2010). Control and interference in task switching-a review. Psychol. Bull. 136, 849–874. 10.1037/a0019842 20804238

[B6] Meier C. Lea S. McLaren I. (2016). Task-switching in pigeons: associative learning or executive control? J. Exp. Psychol. Anim. Learn. Cogn. 42, 163–176. 10.1037/xan0000100 27054382

[B7] Monsell S. Sumner P. Waters H. (2003). Task-set reconfiguration with predictable and unpredictable task switches. Mem. Cognit. 31, 327–342. 10.3758/BF03194391 12795475

[B8] Soto F. Wasserman E. (2010). Error-driven learning in visual categorization and object recognition: a common-elements model. Psychol. Rev. 117, 349–381. 10.1037/a0018695 20438230 PMC2930356

[B9] Stoet G. Snyder L. (2004). Single neurons in posterior parietal cortex of monkeys encode cognitive set. Neuron 42, 1003–1012. 10.1016/j.neuron.2004.06.003 15207244

[B10] Stoet G. Snyder L. (2009). Neural correlates of executive control functions in the monkey. Trends Cogn. Sci. 13, 228–234. 10.1016/j.tics.2009.02.002 19362512 PMC2825374

[B11] Stoet G. Snyder L. H. (2003). Executive control and task-switching in monkeys. Neuropsychologia 41, 1357–1364. 10.1016/S0028-3932(03)00048-4 12757908

[B12] Stoet G. Snyder L. H. (2007). Extensive practice does not eliminate human switch costs. Cogn. Affect. Behav. Neurosci. 7, 192–197. 10.3758/CABN.7.3.192 17993205

[B13] Vandierendonck A. Liefooghe B. Verbruggen F. (2010). Task switching: interplay of reconfiguration and interference control. Psychol. Bull. 136, 601–626. 10.1037/a0019791 20565170

